# Biomechanical and physiological differences between synchronous and asynchronous low intensity handcycling during practice-based learning in able-bodied men

**DOI:** 10.1186/s12984-020-00664-8

**Published:** 2020-02-24

**Authors:** Cassandra Kraaijenbrink, Riemer J. K. Vegter, Alexander H. R. Hensen, Heiko Wagner, Lucas H. V. van der Woude

**Affiliations:** 1 Centre for Human Movement Sciences, University of Groningen, University Medical Centre Groningen, Antonius Deusinglaan 1, 9713 AV Groningen, the Netherlands; 2 Department of Motion Science, Institute of Sports Science, University of Münster, Horstmarer Landweg 62b, 48149 Münster, Germany; 3 Centre for Rehabilitation, University of Groningen, University Medical Centre Groningen, Hanzeplein 1, 9713 GZ Groningen, the Netherlands

**Keywords:** Cyclic exercise, Crank mode, Practice, Efficiency, Force production, Motor learning

## Abstract

**Background:**

Originally, the cranks of a handcycle were mounted with a 180° phase shift (asynchronous). However, as handcycling became more popular, the crank mode switched to a parallel mounting (synchronous) over the years. Differences between both modes have been investigated, however, not into great detail for propulsion technique or practice effects. Our aim is to compare both crank modes from a biomechanical and physiological perspective, hence considering force and power production as a cause of physiological outcome measures. This is done within a practice protocol, as it is expected that motor learning takes place in the early stages of handcycling in novices.

**Methods:**

Twelve able-bodied male novices volunteered to take part. The experiment consisted of a pre-test, three practice sessions and a post-test, which was subsequently repeated for both crank modes in a counterbalanced manner. In each session the participants handcycled for 3 × 4 minutes on a leveled motorized treadmill at 1.94 m/s. Inbetween sessions were 2 days of rest. 3D forces, handlebar and crank angle were measured on the left hand side. Kinematic markers were placed on the handcycle to monitor the movement on the treadmill. Lastly, breath-by-breath spirometry combined with heart-rate were continuously measured. The effects of crank mode and practice-based learning were analyzed using a two way repeated measures ANOVA, with synchronous vs asynchronous and pre-test vs post-test as within-subject factors.

**Results:**

In the pre-test, asynchronous handcycling was less efficient than synchronous handcycling in terms of physiological strain, force production and timing. At the post-test, the metabolic costs were comparable for both modes. The force production was, also after practice, more efficient in the synchronous mode. External power production, crank rotation velocity and the distance travelled back and forwards on the treadmill suggest that asynchronous handcycling is more constant throughout the cycle.

**Conclusions:**

As the metabolic costs were reduced in the asynchronous mode, we would advise to include a practice period, when comparing both modes in scientific experiments. For handcycle users, we would currently advise a synchronous set-up for daily use, as the force production is more effective in the synchronous mode, even after practice.

## Background

Individuals, who use a manual wheelchair for daily locomotion depend on their upper body for mobility and other activities of daily living. Self-propelled hand-rim wheelchairs are often used, as they are especially useful indoors, because of their small size and good maneuverability [1]. However, with a mechanical efficiency (ME) of 5–10% at submaximal level, hand-rim wheelchairs are inefficient and physiologically straining for longer distances, especially outside [2–5]. A more energy efficient upper body exercise mode is arm cranking or handcycling (ME = 10–17% at submaximal level) [2, 3, 6–10]. A practical alternative for outdoor wheeled mobility is therefore the attach-unit handcycle, a crank system that can be attached to/or mounted in front of the hand-rim wheelchair. It makes daily handcycling feasible for a wider public. Especially in a flat country like the Netherlands, where able-bodied individuals use a bicycle for commuting and cycling facilities are optimal, the attach-unit handcycle is a good alternative to go shopping, go to work, school or the sports club, meet with friends, etc. This increases an individual’s independence in daily living and participation in the society following the conceptual framework of the International Classification of Functioning [11].

As the vehicle mechanics of the handcycle originally stem from bicycle technology, the handcycling crank mode was initially asynchronous, i.e. the cranks were mounted with a phase shift of 180 degrees. Over the years, handcycling became more popular and the crank mode switched from asynchronous (Asyn) to synchronous (Syn), in other words to the parallel crank setting seen today [12]. The differences between both crank modes have been subject to research over the years, but to date a proper one-to-one biophysical comparison, combining a biomechanical with a physiological analysis, is lacking. Nonetheless, some work has been performed essentially along two lines of research. Firstly, research has been done with a fixed (arm-crank) ergometer or a handcycle-ergometer, where the handcycle is part of the set-up. Secondly, treadmill based research, where the handcyclist is less constrained and steering is a necessity, has been performed.

During arm cranking, i.e. with an ergometer where the system has no steering option, no effects of crank mode on the physiological responses, like heart rate, oxygen uptake, ventilation and blood lactate levels were found at several sub-maximal power output levels (< 100 W) for able-bodied persons [13, 14], wheelchair users [14] as well as for experienced handcyclists [10]. However, with this experienced group, a higher mechanical efficiency is found for the asynchronous mode (16–17%) over the synchronous (14–15%) [10]. In a more biomechanical approach with a handcycle-ergometer set-up, Faupin et al. (2011) found more range of motion of the trunk, shoulder and elbow in the asynchronous mode. The torque produced in the synchronous mode was significantly lower during the pull phase and significantly higher during the push up than during asynchronous handcycling, indicating a different propulsion style between modes [15].

On the other hand, for submaximal handcycling with an attach-unit handcycle on a motorized treadmill, where steering is necessary, a number of studies in able-bodied men showed that the asynchronous mode is physiologically less efficient than synchronous handcycling. Mechanical efficiency is lower (− 1–2%) and oxygen uptake (maximal + 200 mL/min), ventilation (maximal + 10 L/min) and heart rate responses (maximal 20 bpm or 15% %HRR) were reported higher in submaximal asynchronous steady state handcycling [9, 16–18]. This was found across different cadences [9, 17], treadmill speeds [17, 18], treadmill slopes [9, 16], and power output levels [16]. In addition to these physiological parameters, Bafghi et al. (2008) was one of the first to measure the muscle activity and the external forces at the handlebar [18]. The muscles that stabilize the crank system were found to be activated more in the asynchronous mode, whereas muscles around the shoulder showed higher activation in the synchronous mode. Higher mean forces in the sagittal plane were seen during synchronous handcycling, whereas no significant differences in the mediolateral forces were found. Unfortunately, the tangential (propulsion) force and the radial force components could not be differentiated in their set-up. Although this research is the first to consider biomechanics, the differences between both crank modes have not been investigated with a three-dimensional analysis. Building on the initial insights from Bafghi et al., a 3D approach is used in the current study, adding the possibility to differentiate between steering and power producing forces. As asynchronous handcycling was found to be more physiological straining than synchronous, it was suggested that the asynchronous mode is inherently less stable when propelling forward on the treadmill, because one needs to combine steering and power production, where the latter tends to destabilize coasting direction [16–18]. With our 3D approach, force and power production can serve as a cause for the physiological measures and the knowledge on the differences between crank modes can be amended.

To the authors’ knowledge, the effects of the crank mode are limited to aforementioned cross-sectional study designs in which the participant ride once in every crank mode. To date, short-term practice effects on efficiency and technique are not investigated in handcycling. Yet, one of the characteristics of a skilled motor performance is an optimization of energy expenditure [19–21]. With practice, novices are able to reduce physiological costs, as was found for walking, creeping [20] and race-walking [22] amongst others. For rowing, a closed-chain cyclic movement, like handcycling, similar results have been shown. Within ten practice sessions of 16 min in a rowing ergometer, measures of rating of perceived exertion, as well as the oxygen consumption significantly decreases (− 2.5 ml/kg/min), as economy increases (+ 0.4 W/ml) [21]. Besides these physiological measures, rowing technique improves, as represented by reductions in stroke rate, peak force variability and muscle activation. These results support the hypothesis that a strong link exists between physiology and movement coordination. In addition, for forms of wheeled mobility, other than handcycling, it has been shown that natural motor learning takes place after a short period of practice [5, 23–25]. For instance, mechanical efficiency and propulsion technique (e.g. frequency and stroke angle) can improve even within 12 min of low intensity hand-rim wheelchair propulsion [23]. For handcycling, both mechanical efficiency and cycling technique, i.e. the force application pattern, are to be expected to improve over time. Thereupon, one of the objectives of this study was to include the process of natural motor learning within the biophysical approach towards the comparison of both modes of handcycling, shining a new light on the matter.

In the current pre-test and post-test study, the differences between synchronous and asynchronous handcycling at low intensity were systematically evaluated. Able-bodied novices were assessed during practice-based learning on a motor driven treadmill by taking a biophysical approach. It was hypothesized that the physiological response is initially higher for asynchronous handcycling than for synchronous handcycling, in line with previous research. However, canceling out the instability in asynchronous handcycling is a skill that is expected to be learned over time, whereas this instability does not exists for synchronous handcycling. An increase in mechanical efficiency and force effectiveness in asynchronous handcycling are likely the result of practice. Therefore, the difference between both modes should reduce with practice.

## Methods

### Participants

Twelve able-bodied men (age: 23.9 ± 1.2 years, mass: 78.6 ± 9.1 kg, height: 1.81 ± 0.05 m and arm length: 0.64 ± 0.02 m) volunteered to take part in this low intensity handcycle study after being given written and verbal information and signing an informed consent form. Exclusion criteria were having shoulder complaints or impairments, having any medical conditions (PAR-Q [26]), or having any handcycling experience. The latter, to ensure an equal experience level in both modes across participants. The local ethics committee of the Centre for Human Movement Sciences, University Medical Centre Groningen, University of Groningen, the Netherlands approved the study (No. ECB/2015.06.17_1).

### Set-up and protocol

**Fig. 1:**
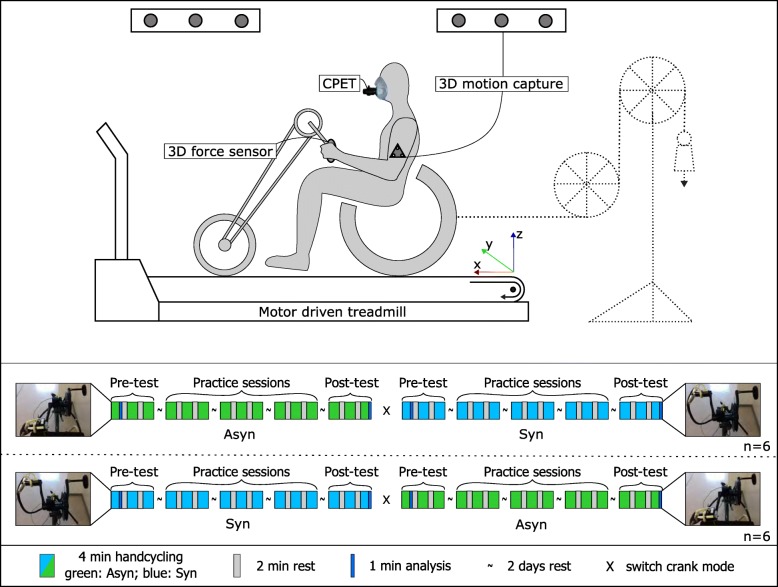
Overview of the experimental set-up (top) and the cross-over practice protocol for synchronous (Syn) and asynchronous (Asyn) handcycling (bottom). Top: The participants, equipped with a mask and heart rate monitor for cardiopulmonary exercise testing (CPET), rode in the attach-unit experimental handcycle with kinematic markers on the motor driven treadmill at 1.94 m/s. During the practice sessions, a pulley system was attached to the back of the handcycle to impose resistance [32]. Bottom: The experimental protocol consisted of a pre-test, three practice sessions, and a post-test, all structured in three blocks of 4 min of exercise with 2 min rest in between. After completion the protocol was repeated for the other crank mode (Syn/Asyn). In between sessions was a two-day break (~). The fourth and very last minute of exercise in each crank mode were analyzed to compare the effects of crank mode and motor learning

During the complete experiment, the participants rode on a level motor driven treadmill (2.4 × 1.2 m, Motekforce Link b.v., Amsterdam, the Netherlands) at 1.94 m/s, as is within the range found suitable for handcycling [16, 18, 27]. The experiment consisted of a pre-test, followed by three practice sessions and a post-test in one of the two crank modes, followed by the same line-up of activities in the other mode (Fig. 1). The order of crank mode was counterbalanced, i.e. half of the participants started with the synchronous mode, half with the asynchronous mode. For each mode, each of the five 16 min sessions consisted of three four-minute blocks with two minutes rest in-between. In the resting period, Rate of Perceived Exertion (RPE; Borg Categorical 6–20 Scale [28]) was registered to check the sub-maximal conditions. To ensure enough rest between sessions, the participants came back every 3 days. The pre- and post-test consisted of level handcycling without additional resistance (~ 15 W) at 60 rpm [9, 17, 29]. In the three practice sessions, resistance (+ 0 W, + 10 W, + 20 W) and cadence (52, 60, 70 rpm) were varied as part of a bigger research project [30, 31]. To ensure a physiological steady state, only the last minute of the selected four-minute blocks were analyzed with Matlab (MATLAB 2018b, MathWorks Inc., Natick, Massachusetts, USA).

### Instrumented handcycle and kinetic measures

All participants used the same instrumented add-on handcycle, that was custom made by the Technical Support Workshop of the Faculty of Behavioral and Movement Sciences of the VU University Amsterdam; see van Drongelen et al. (2011) for specifications [33]. No individual seating adjustments were made within this experiment. The crank axis height was just below the acromion for all participants. The handcycle had a seven-speed hub gear (Shimano Inter 7 SG-7C18, Shimano Inc., Osaka, Japan) from which the second gear (gear ratio: 0.741) was used during pre- and post-test. The front wheel was 16 in. and had a tire pressure of 260 kPa, the rear wheels were 24 in. and had a tire pressure of 600 kPa. The crank length was 0.17 m. The crank mode was changed by demounting the right crank, rotating the left crank 180°, and remounting the right crank. The start of the propulsion cycle was defined as a horizontal crank position closest to the participant.

**Fig. 2:**
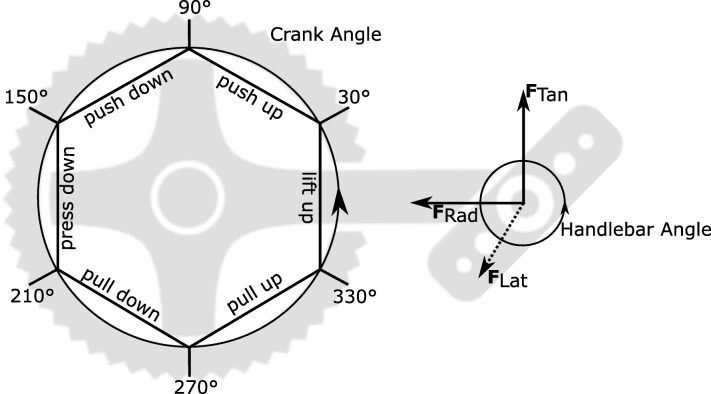
Definition propulsion phases [34] and coordinate system of the forces, as seen from the left side (i.e. coasting direction right-left; rotation counterclockwise)

Only the left handlebar of the handcycle was equipped with a 3D force transducer (100 Hz, AMTI, Watertown, MA, USA) and two optical encoders (Type 19, Elcis, Collegno, Italy) that recorded the handlebar and the crank angle. The force components, mediolateral (**F**_Lat_, N), radial **(F**_Rad_, N) and tangential force **(F**_Tan_, N), were defined in the local coordinate system of the handlebar based on the handlebar angle (Fig. 2). Consequently, the resultant force was calculated. Kinetic data was low pass filtered (2nd order Butterworth, cut-off frequency 5 Hz, sample frequency 100 Hz) and resampled to one data point per degree using ‘interp1’ in Matlab. The average cycle of all complete cycles of the last minute was calculated for all force components.

The propulsion cycle was divided in six phases; push up (30–90°), push down (90–150°), press down (150–210°), pull down (210–270°), pull up (270–330°), and the lift up phase (330–30°) [27, 34, 35].

The angular velocity of the crank (**ω,** rad/s) was determined as the first derivative of the crank angle. The external power output produced at the handle was calculated according to eq. 1.

1 $$ {P}_{External}(W)={\boldsymbol{F}}_{Tan}\bullet \boldsymbol{\omega} \bullet {Length}_{crank} $$

**Fig. 3:**
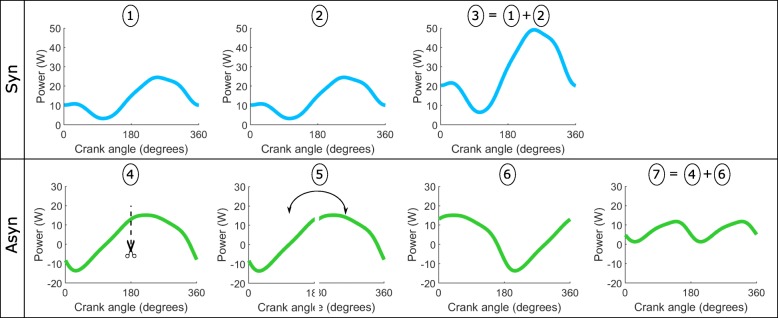
Procedure to calculate the external power output of both hands (example for one cycle). The top figures show the procedure for synchronous handcycling. (1) The external power output as measured at the left handlebar. (2) The assumed external power output produced at the right handlebar, i.e. equal to the left. (3) The total external power output as the sum of both hands. The bottom figures show the procedure for the asynchronous mode. (4) The external power output as measured at the left handlebar. As the right handlebar is mounted with a 180 degree shift, this data is ‘cut’ at 180 degrees. (5) The last 180 degrees is placed in front of the first 180 degrees of the cycle. (6) The result of step 5, i.e. the assumed external power output at the right handlebar. (7) The total external output as the sum of both hands

Since the right side was not instrumented, the total power produced by both hands was assumed to be on average similar, since participants had to drive in a straight line on the treadmill [27]. For synchronous handcycling, this means that the power produced at both handles is the measured power output times two. For the asynchronous mode, we took the measured power output, applied a phase shift of 180° and added this to the original measured power output. In Fig. 3, this procedure is shown for one cycle. The average cycle of all complete cycles of the last minute was calculated for further analysis.

The efficiency of force application at the handlebar is calculated for every time step as the fraction of effective force (FEF, %) according to eq. 2 [36].

2 $$ FEF\ \left(\%\right)=\frac{{\boldsymbol{F}}_{Tan}}{{\boldsymbol{F}}_{Resultant}}\ast 100\% $$

The mean FEF for both hands was determined. This procedure was similar to the calculation of external power output. For synchronous handcycling, we assumed that the FEF at the right handlebar was equal to the one measured at the left. The total FEF was the mean value of both hands. For asynchronous handcycling, we performed the same 180 degree phase shift as shown in Fig. 3 (step 4–6). The measured FEF of the left side was added to these assumed values on the right side. Thereupon, the added values were divided by two to gain the mean value.

### Kinematic measurements

The 3D position of the handcycle with respect to the treadmill was determined with the use of an optoelectronic camera system (Optotrak, Northern Digital, Waterloo, Canada) and two cluster markers (3 active markers each) at a frequency of 100 Hz. One cluster was placed on the crank system and one on the wheelchair. The data from these two clusters were transformed into six virtual markers, namely the front wheel axis left and right, the crank axis left and right, and the rear wheel axes left and right. Missing data was fitted with a spline method using the function ‘fillmissing’ in Matlab.

The distance travelled in the forward-backwards direction (x, Fig. 1) and from left to right (y, Fig. 1) was determined for each marker according to eq. 3. The total distance travelled by the handcycle relative to the treadmill position was defined as the average value of all six markers.

3 $$ Distance\ (m)= sum\left( abs\left( diff(Position)\right)\right) $$

### Physiological measurements

A breath-by-breath gas exchange data analyzer with heart rate sensor (Cosmed Quark CPET, Cosmed, Rome, Italy, via TulipMed, Nieuwegein, the Netherlands) continuously measured oxygen uptake (VO_2_, mL/min/kg), carbon dioxide output (VCO_2_, mL/min/kg), ventilation (VE, L/min/kg), breathing frequency (BF, breaths/min), and heart rate (HR, beats/min). The system was regularly calibrated using a 16% O_2_, 5% CO_2_ calibration gas, as well as using a certified 3-L calibration syringe before every session. Energy expenditure (EE, W) was calculated using VO_2_ and VCO_2_ according to eq. 4 [37].

4 $$ EE(W)=\left(\frac{\left(4.94\ast \frac{V{CO}_2}{V{O}_2}+16.04\right)\ast {VO}_2}{60}\right) $$

Subsequently, mechanical efficiency (ME, %) was calculated according to eq. 5 [37].

5 $$ ME\ \left(\%\right)=\frac{mean\left({P}_{External, both\ hands}\right)}{mean\left( Energy\ Expenditure\right)}\ast 100\% $$

### Statistics

A two-way repeated measures ANOVA was performed for all twelve participants with the within subject factors ‘crank mode’ and ‘practice’. The effect of crank mode was defined as the comparison of synchronous versus asynchronous handcycling. The effect of practice was defined as the comparison of the final minute of the first block of the pre-test, i.e. the fourth minute of handcycling, versus the last minute of the third block of the post-test, i.e. the last minute of handcycling in a given mode.

For the kinetic data, the mean cycle of all participants, i.e. the force profile, was determined. The 1D SPM method was used to analyze the force profiles using the function ‘anova2rm’ with a significance level of α = 0.05 (spm1d package for Matlab, Pataky 2018 [38]).

For kinematic and physiological data mean values were calculated for every variable, before including them in the statistical analysis in SPSS (IBM Corp. Released 2017. IBM SPSS Statistics for Windows, Version 25.0. Armonk, NY: IBM Corp). Two kinematic measurements are missing, due to technical problems. The mean of the other participants were taken for the missing values of that condition. Kinematic data was not found to be normally distributed. Therefore, two Wilcoxon Signed Ranks Tests were performed, one to compare synchronous versus asynchronous handcycling (*n* = 24, independent of practice), one to test the effect of practice (*n* = 24, independent of mode). The significance level (2-tailed) was corrected for multiple tests according to Bonferroni, hence α = 0.025.

The rate of perceived exertion also was not found to be normal, therefore two Wilcoxon Signed Ranks Tests were performed as well (α = 0.025). The physiological data was found to be normally distributed with the Shapiro-Wilk test and the ANOVA could be performed with a significance level of α = 0.05.

## Results

### Kinetic effects

**Fig. 4:**
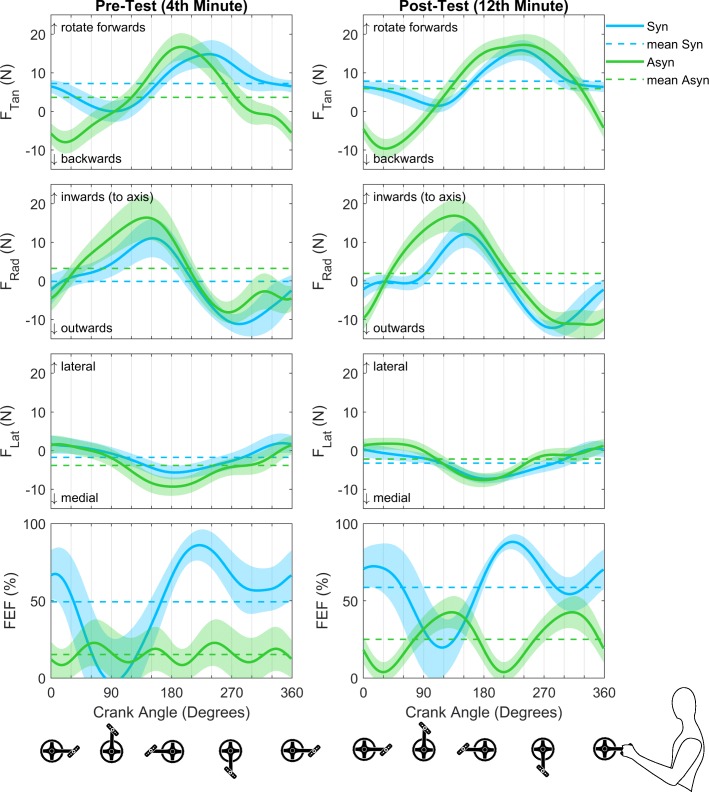
Unilateral force profiles of the three force components (**F**_Lat_, **F**_Rad_, and **F**_Tan_) and the bilateral fraction of effective force (FEF) profile of the 4th minute of the pre-test (last minute 1st block, left column) and last minute of the post-test (last minute 3rd block, right column). The mean cycle (± SD) of all participants (*n* = 12) as well as the time-averaged values (−-) are shown for both crank modes

**Fig. 5:**
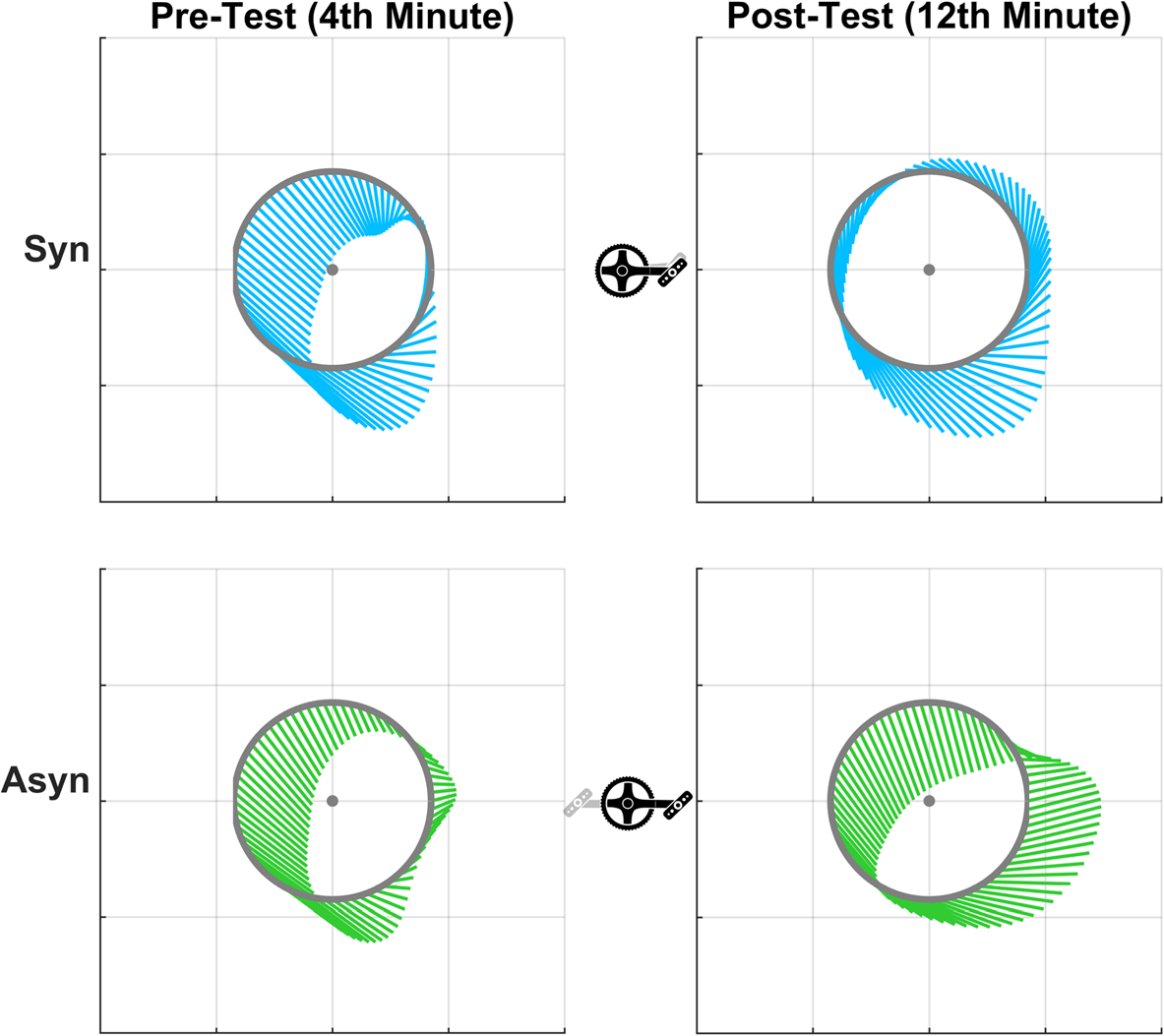
Unilateral force production (shown per 5°) in the local sagittal plane of the crank set (resultant **F**_Tan_ and **F**_Rad_). The average overall cycle of the 4th minute of the pre-test (last minute 1st block) and last minute of the post-test (last minute 3rd block) were shown for both crank modes (blue = synchronous; green = asynchronous) for one participant. The force data was low-pass filtered (2nd order Butterworth, cut-off frequency 5 Hz, sample frequency 100 Hz)

In terms of force production, a difference between both crank modes is found for the three force components (F_tan_, F_rad_, F_lat_), as well as FEF (Fig. 4). The tangential force, i.e. the propulsive force component, is significantly higher in the synchronous mode during the lift/push up phases (F*(1,11) = 13.06; *P* = 0.018 for 325–360°; *P* < 0.001 for 1–80°). This force is higher in the asynchronous mode for the push/press down phases (*P* = 0.001 for 132–201°). The radial force is lower in the synchronous mode than in the asynchronous (Figs. 4 and 5), in particular during the lift up (F*(1,11) = 13.40; *P* = 0.044 for 349–360°; *P* = 0.036 for 1–19°) and the push up/down phases (*P* < 0.001 for 46–151°). The mediolateral force component shows differences in the push/press down phases (F*(1,11) = 11.31; *P* = 0.006 for 126–209°). The FEF of both hands is higher in the synchronous mode for most of the cycle, except for during the push up/down phases (F*(1,11) = 13.56; *P* = 0.001 for 1–58°; *P* < 0.001 for 161–360°).

With practice, no differences were seen in the force effectiveness or the mediolateral force production, however, a significant difference in the force production in the sagittal plane is seen (Fig. 4). A small shift and increase in the tangential force production is seen, as a significant effect (*P* < 0.001) is found in the pull down/up phases (*P* < 0.001 for 248–342°). The radial force component is changed in the pull/lift up phases (*P* < 0.001 for 288–360°; *P* = 0.034 for 1–21°). Especially in the synchronous mode, a decrease is found with practice.

For FEF (*P* = 0.007 for 28–69°), the tangential (*P* = 0.016 for 50–87°) and mediolateral force production (*P* = 0.039 for 57–85°) an interaction effect of practice and crank mode during the push up phase is found. In addition, interaction effects during the pull down/up phases are found for the tangential (*P* = 0.001 for 254–325°) and mediolateral force production (*P* = 0.002 for 200–298°).

### Power production

**Fig. 6:**
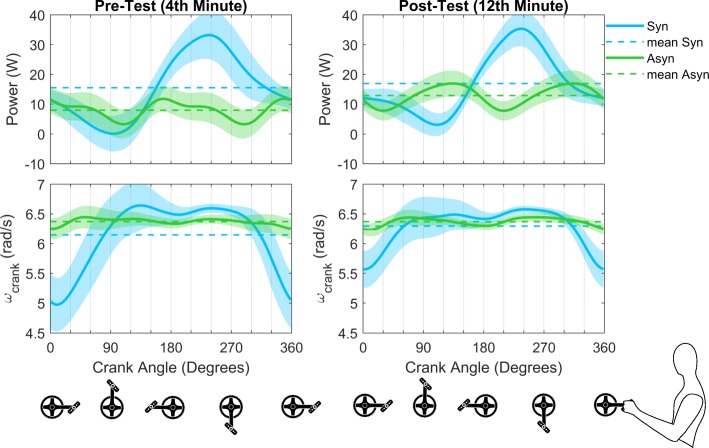
Mean cycle (± SD) of all participants (*n* = 12) of the external power output (as a result of both hands) and the crank’s rotation velocity are shown as profile plots. Time-averaged values (−-) per crank mode are also shown

Surprisingly, the calculated external power output in the same handcycle, subject group and during identical treadmill speed is significantly different for both crank modes (Fig. 6). A more constant power output is seen in the asynchronous mode compared to the synchronous mode, where less power is produced in the push down phase (F*(1,11) = 13.41; *P* = 0.006 for 94–139°) and more in the press down, pull down/up phases (*P* < 0.001 for 165–292°). The crank velocity reflects this continuity as well (Fig. 6). The velocity is practically constant in the asynchronous mode, whereas two stages (speed up/slow down) can be recognized in the synchronous mode. The crank’s rotation is slower during the lift/push up (F*(1,11) = 16.19; *P* < 0.001 for 324–360° and 1–55°). The crank is faster during the press/pull down phase (*P* < 0.001 for 183–287°).

In the pre-test a similar reduction in power output during the push phases is seen for both crank modes. In the post-test, however, an increase in power output is seen in the asynchronous mode, because of practice (*P* = 0.007 for 71–115°). A practice effect is also found in the pull phases (*P* = 0.003 for 266–318°), due to a change in the power output production in the asynchronous mode. For the synchronous mode, the decrease in crank velocity during the lift/push up phases is less after practice (*P* = 0.019 for 270–285°) and a more constant crank rotation is seen (*P* = 0.042 for 202–208°). In addition, in the push down phase an interaction effect on the power output is found (*P* = 0.040 for 117–131°). For crank velocity, interactions in the push up (*P* = 0.040 for 39–46°) and pull down phases (*P* = 0.002 for 237–267°) are found.

### Kinematic effects

**Fig. 7:**
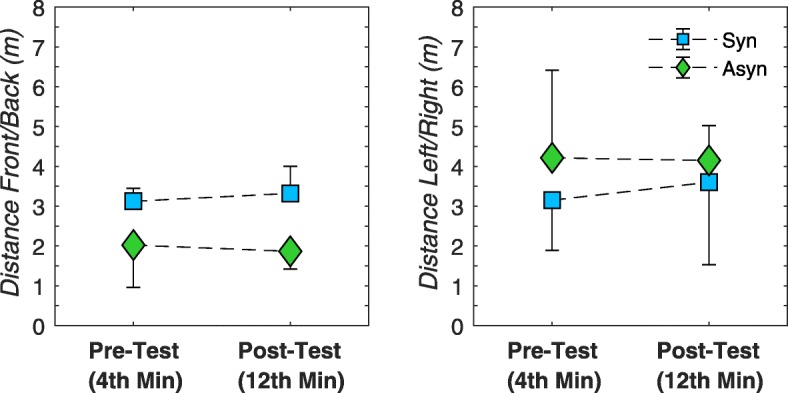
Mean (± SD) of all participant (*n* = 12) for kinematic variables represented as the average value over the last minute of synchronous (blue square) and asynchronous (green diamond) handcycling

The Wilcoxon Signed Ranks Test to compare both modes revealed that the participants travelled significantly less back and forwards on the treadmill in an asynchronous handcycling mode compared to the synchronous mode (z = − 3.80, *P* < 0.001, *r* = − 0.78). In addition, no other significant differences were found for travelled distances, hence no practice effects were found. (Fig. 7).

### Physiological effects

**Fig. 8:**
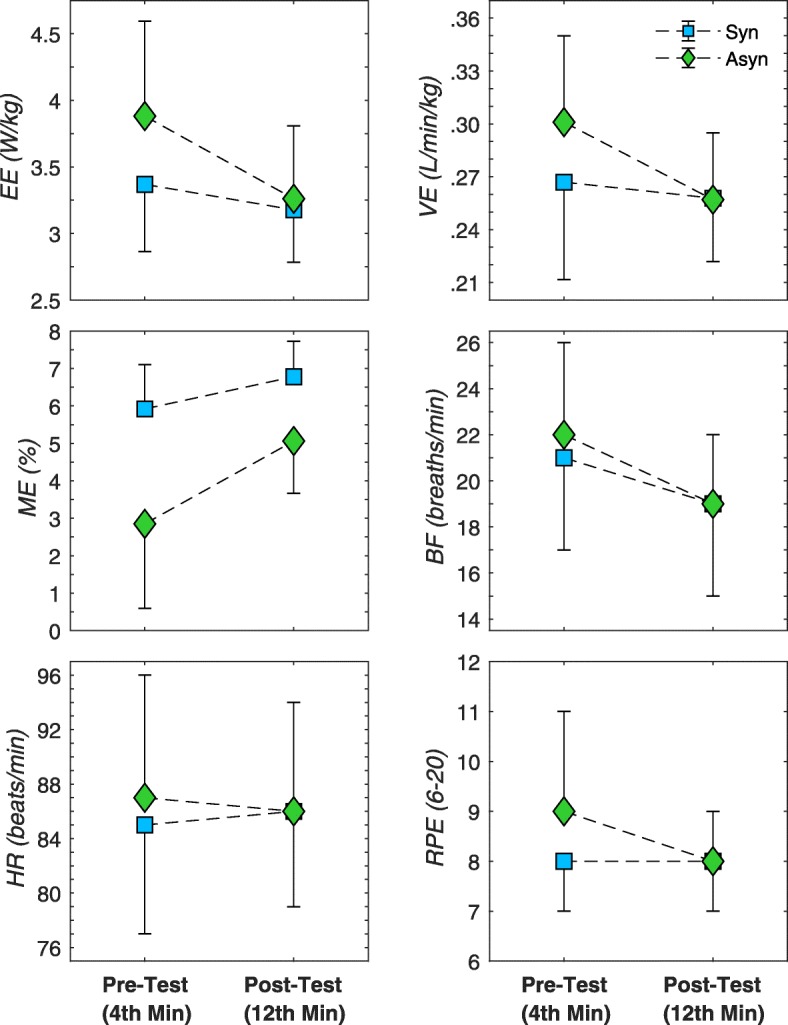
Mean (± SD) of all participant (*n* = 12) for physiological variables represented as the average value over the last minute of synchronous (blue square) and asynchronous (green diamond) handcycling

As shown in Fig. 8, a difference in metabolic response to synchronous and asynchronous handcycling can be found, especially at the pre-test. A significant effect of crank mode was found for mechanical efficiency (F(1,11) = 17.71, *P* = 0.001, η_p_^2^ = 0.62) and energy expenditure (F(1,11) = 6.20, *P* = 0.030, η_p_^2^ = 0.36). For the other variables no significant effects were found.

Practice reduces metabolic cost, especially in the asynchronous handcycling mode. A significant effect of practice was found for mechanical efficiency (F(1,11) = 42.71, *P* = < 0.001, η_p_^2^ = 0.78), energy expenditure (F(1,11) = 45.79, *P* < 0.001, η_p_^2^ = 0.81), ventilation (F(1,11) = 12.35, *P* = 0.005, η_p_^2^ = 0.53) and breathing frequency (F(1,11) = 13.94, *P* = 0.003, η_p_^2^ = 0.56). No significant effects on heart rate or RPE were found.

## Discussion

Without any handcycle experience, asynchronous handcycling is indeed less efficient than synchronous handcycling in terms of physiological strain, handcycle force effectiveness and power production. After practice, however, equal values in metabolic cost are found for both crank modes, due to a reduction in the asynchronous mode. The force production is more efficient in the synchronous mode, as remains the case after practice. However, greater learning effect seems apparent in the asynchronous mode. From external power production, crank rotation velocity and the distance travelled front to back on the treadmill, it becomes clear that asynchronous handcycling is more constant throughout the cycle.

### Effects of crank modus

**Fig. 9:**
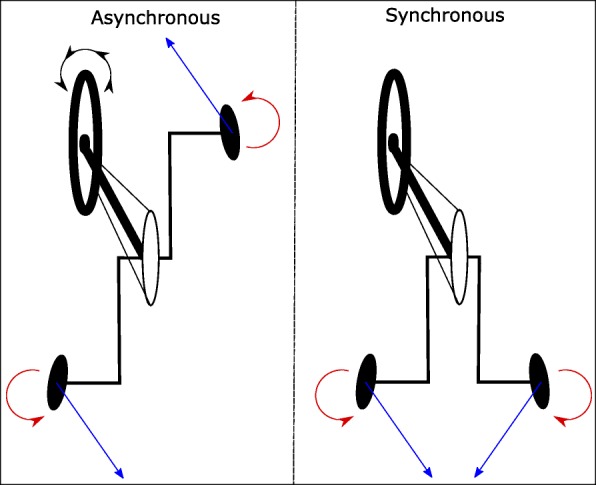
Example of the effects of force production on the handlebars on the steering moments and the rotation of the front wheel during asynchronous (left) versus synchronous (right) handcycling. Asynchronous: The depicted forces (blue arrows), e.g. both tangential, will both cause a steering moment in the counterclockwise direction (red), and consequently the front wheel will turn in the same direction. Synchronous: The depicted forces (blue), both tangential, will cause an opposite moment around the crank steering axis, in this case a counterclockwise rotating moment on the left and a clockwise rotating moment on the right (red). These moments cancel out each other, hence they cause no rotation of the front wheel

The handcycle movement is very complex, as the force production at the handlebars causes two moments, one around the crank axle for propulsion and one around the front fork axle for steering. The user wants to increase the total moment around the crank axle, while reducing the moment around the steering axle. A more tangential force would increase the moment around the crank axle for both modes. However, the steering moment at the left and right handlebar would be in the same direction for the asynchronous mode, causing a rotation of the front wheel, whereas the steering moments would cancel out in the synchronous mode (Fig. 9). Therefore, more control is needed in the asynchronous mode, which should be reflected in the direction of the force produced at the handlebar. It seems that the participants are quite capable to do this, as no significant difference in left-right movement on the treadmill was found.

Compensation strategies to keep the front wheel from rotating seems to take place in the sagittal plane, as we find large differences in the tangential and radial force components and only small differences in the mediolateral force component between both modes. Our results are in line with earlier findings, as Bafghi et al. (2008) reported a difference in the mean total force produced in the sagittal plane, but no differences in the mediolateral force. The latter forces seem to be out-regulated by co-activation of the muscles in trunk and arms that stabilize the crank system [18]. In the current study, large pulling forces in order to propel the handcycle are found for both modes. In the asynchronous mode, however, a negative tangential force is found in the pull up, lift up and push up phases. As a phase shift of 180° exists in the crank angle for the right hand, the right hand is in the phases where large propulsion force is produced. Thus, the large negative tangential force could be a compensation of the instability (steering moment) caused by the propulsion. In addition, it seems that one pulls the cranks towards the chest to control turning, as the radial force is positive from 30 to 210° and negative for 210–30°, as shown in Figs. 4 and 5. Because of these compensation strategies, the effectiveness of force production is less in the asynchronous mode. Especially after practice, the difference in propulsion style between modes becomes clear, as represented in the graphs on the right of Fig. 5.

Surprisingly, we found a difference in the power production between both modes (pre-test: Syn: 15.5 W vs Asyn: 7.3 W; post-test: Syn: 16.9 W vs Asyn: 12.9 W), even when the treadmill was leveled and set at 1.94 m/s for both conditions. One cause might be the difference in the forward-backwards movement on the treadmill. In the synchronous mode, the push phases of the cycle are not efficient, as represented by FEF and the power production. A large pulling force/power production is found, during which the crank velocity increases. It can be assumed that after this acceleration, the crank decelerates, because of the weight of the arms and hands holding the handles, not actively rotating the cranks. This means that participants did not exactly drive 1.94 m/s, but showed an acceleration in the pulling phases and a deceleration in the pushing phases, as was earlier reported for synchronous handcycling [39]. In the asynchronous mode, these acceleration-deceleration phases are not apparent. A more constant propulsion movement is seen in the asynchronous mode, shown by the fact that the power production is better distributed over the cycle, the crank velocity and the forward-backwards movement of the entire handcycle are more constant. However, at the same treadmill speed, this resulted in lower external power output values for the asynchronous mode. As the EE is the same after practice, one could say that synchronous is more efficient, as more power can be produced with the same amount of metabolic cost, as represented by ME.

### Effects of practice

The results of the pre-test are comparable to previous research with a similar set-up, i.e. a handcycle on a treadmill [9, 16–18]. During the pre-test, the synchronous mode shows favorable outcomes over the asynchronous mode with respect to the metabolic costs and force effectiveness. On the other hand, the results show that the difference in metabolic costs is no longer present after practice. As the practice time was too short to evoke physiological training effects, this reduction in metabolic cost might indicate motor learning, as was previously shown for other movements [20–22, 24, 25]. As a cause of the difference between modes, it was suggested that co-activation of the upper body muscles takes place in the asynchronous mode, as one needs to keep the arms in the sagittal plane and therefore reduce the rotation of the trunk [9, 17, 18]. In the synchronous mode the trunk’s inertia can be used for the forward propulsion. It seems that before practice, i.e. at the pre-test, the same propulsion style is used for both cycling modes, as represented by force production in the sagittal plane, as shown in the graphs on the left of Fig. 5. The change in the force production pattern, might be another indication of motor learning within this short practice protocol. The small changes in the external force and power production may lead to a reduction of co-activation of the upper body muscles. This could be the cause of the reduction in metabolic costs. To confirm this hypothesis, electromyography measurements of the upper body are needed in future experiments.

### Limitations

The current experiment was conducted with able-bodied participants and may not represent the population of wheelchair users. Yet this was intended, as novices in both crank modes were needed to simulate early rehabilitation, in which natural motor learning takes place and could help to understand the underlying biomechanical principles regarding crank mode. In addition, an arm-powered propulsion technique was adopted, in which the trunk is not involved in the propulsion. Therefore, it is more likely that wheelchair users would be able to show similar results. To confirm our results, however, practice effects should be investigated in this population as well.

For wheelchair propulsion, Vegter et al. (2014) showed that the groups of novices could be divided into initially fast and initially slow improvers [4]. In the current project, we did not include these inter individual differences in improving and only analyzed the practice effects on group level. As the main focus was not on inter individual differences, the number of participants were too low for such an analysis.

In our analysis, it was assumed that both hands produce the same amount of force, simultaneously in synchronous and with a 180° shift in the asynchronous mode. However, Verellen et al. showed that this was not the case for a handcycle user with spinal cord injury (level C5–6) as different force profiles for both hands were found [40]. To confirm the changes in force/power profiles found in the current study, one needs to measure the forces at both handlebars.

No conclusive explanation about the causes of the reduction of the metabolic costs could be given by the force and power production solely. Without electromyography measurements, assumptions about co-activation in the arms had to be made.

## Conclusion

Without practice, low intensity synchronous handcycling is found to be more efficient than asynchronous handcycling in terms of force effectiveness, mechanical efficiency and metabolic cost. With practice, a great physiological improvement is seen in asynchronous handcycling, whereas synchronous handcycling showed a small increase in efficiency as well. As a result, metabolic costs are equal for both crank modes. The force application effectiveness and mechanical efficiency improved in asynchronous handcycling, but are still not as efficient as synchronous handcycling. Based on the results, we would advise to include a practice period, when comparing both these modes in scientific experiments. For individuals who depend on their upper body for their mobility, we would currently advise a synchronous set-up for daily handcycle use. However, with regard to the external power output and speed fluctuations, it seems beneficial to find a solution that helps with a more constant external power output, leading to reduced speed fluctuations. If the unintended steering moments can be cancelled out by the mechanics of the handcycle, asynchronous handcycling could be an option in the future. More research about the influence of steering on handcycling is needed.

## Data Availability

The datasets used in the current study can be provided by the corresponding author on reasonable request.

## Abbreviations

ANOVA: Analysis of variance
Asyn: Asynchronous handcycle mode
BF: Breathing frequency
CPET: Cardiopulmonary exercise testing
EE: Energy expenditure
FEF: Fraction of Effective Force
F_lat_: Mediolateral force component
F_rad_: Radial force component
F_tan_: Tangential force component
HR: Heart rate
ME: Mechanical efficiency
PAR-Q: Physical Activity Readiness Questionnaire
P_External_: External power output
SPM: One-dimensional Statistical Parametric Mapping
Syn: Synchronous handcycle mode
VCO_2_: Carbon dioxide output
VE: Ventilation
VO_2_: Oxygen uptake
